# Quantitative Benefit–Risk Assessment of COVID-19 Vaccines Using the Multi-Criteria Decision Analysis

**DOI:** 10.3390/vaccines10122029

**Published:** 2022-11-27

**Authors:** Kyung-Hwa Son, Sun-Hong Kwon, Hye-Jung Na, Youngsuk Baek, Inok Kim, Eui-Kyung Lee

**Affiliations:** School of Pharmacy, Sungkyunkwan University, Suwon 16419, Republic of Korea

**Keywords:** SARS-CoV-2, COVID-19 vaccine, multi-criteria decision analysis, quantitative benefit-risk assessment

## Abstract

In the early SARS-CoV-2 (COVID-19) pandemic, four major vaccines were approved despite limited efficacy and safety data through short regulatory review periods. Thus, it is necessary to assess the benefit-risk (BR) profiles of the COVID-19 vaccines. We conducted a quantitative BR assessment for four COVID-19 vaccines (mRNA-based: mRNA-1273 and BNT162b2; viral vector-based: Ad26.COV.2 and ChAdOx1-S) using multi-criteria decision analysis. Three benefit criteria and two risk criteria were considered: preventing COVID-19 infection for (1) adults aged ≥18 years; (2) seniors aged 60 years or older; and (3) severe COVID-19, adverse events (AEs), and serious AEs. Data were retrieved from clinical trials, observational studies, and county-specific AE monitoring reports. Based on the collected data, vaccines were scored for each criterion. 22 professionals weighted each criterion. The overall BR score was calculated using scores and weights. mRNA-1273 was the most preferred vaccine in pre-authorization and BNT162b2 in post-authorization. We found that the mRNA vaccine had a good balance between the benefits and risks. Using this BR assessment, the benefit-risk profile of COVID-19 vaccines can be updated with cumulated data. It will contribute to building evidence for decision making by policy makers and health professionals.

## 1. Introduction

In 2019, the emergence of a new coronavirus, SARS-CoV-2 (COVID-19), resulted in more than 607 million cases of COVID-19 infection and 6.5 million COVID-19-related deaths (as of 9 September 2022) [1]. One year after the World Health Organization (WHO) declared COVID-19 a global pandemic, the US Food and Drug Administration (FDA) approved the first COVID-19 vaccine for emergency use on 11 December 2020, following other vaccines approved in the US and Europe [2]. Given the ongoing pandemic, COVID-19 vaccines have been approved based on less evidence than that required for traditional approval regarding the efficacy and safety of vaccines. Regulatory authorities have approved the use of COVID-19 vaccines at extraordinary speed. The average time of emergency use approval review was 21 days in the FDA and the European Medicines Agency because the regulatory authorities applied new pathways and frameworks that accelerated vaccine authorization with limited pre-approval evidence. Additional safety and efficacy data are required to obtain full approval [2].

In 1998, the Council for International Organizations of Medical Sciences (CIOMS) introduced the need for a more systematic and consistent approach to evaluate the benefits and risks of marketed drugs [3]. In addition to the Pharmacoepidemiological Research on Outcomes of Therapeutics by the European Consortium (PROTECT), there have been various contributors providing benefit-risk frameworks and methodological recommendations [4]. A quantitative BR assessment using elicited BR preference can be used to compare the BR profile of a medical product with that of others. Thus, the quantitative approach ensures continuity and consistency across drug BR assessments and makes decisions easier to justify [5]. The IMI-PROTECT Benefit-Risk Group developed the recommendations for the methodology and visualization techniques to assess the benefit and risks of medicines, and suggested multi-criteria decision analysis (MCDA) models as a quantitative BR assessment [6,7,8]. MCDA allows the assessment of a product of interest considering multiple criteria simultaneously and provides a quantitative value for each product, covering benefits and risks [5]. MCDA is a highly effective and flexible framework to integrate multiple benefits and risk criteria with preference values, and use them to compare each alternative option [9,10]. Moreover, breaking down complicated problems makes it easier to see how much risk and benefit are worth. Kurzinger et al. (2020) presented published studies for structured quantitative benefit-risk assessment using MCDA for the BR profile of medicinal products during the early drug development phase or in post-authorization [7]. This method is based on a hierarchical value tree with evaluation criteria for different options. The score for the expected performance of the evaluation criteria is obtained and the weighted preference value for each criterion can be determined by experts. This approach is promising because it identifies the options with the most favorable risk-benefit ratio, which outcome (risk or benefit) is more influential, requires further investigation, and has the most favorable risk-benefit ratio. These values can be used to compare treatments as evidence for decision making. The MCDA has the potential to support health technology assessment agencies in formulating high-quality, consistent, and transparent recommendations [11]. The study applied quantitative BR models based on the generic benefit-risk roadmap proposed by IMI PROTECT [8]. The study was designed to include planning, exploring evidence, collecting data, analyzing those data, and drawing a conclusion. The MCDA process for the assessment of BR was detailed in Materials and Methods.

Currently, there are no studies evaluating a COVID-19 vaccine using quantitative BR assessment, although COVID-19 vaccines have already been used. Since the COVID-19 vaccine has been approved for emergency use with limited requirements, its safety and efficacy should be monitored continuously with cumulative evidence. Changes in the BR profile need to be assessed using evidence before and after approval. Therefore, we aimed to quantitatively evaluate the benefits and risks of the COVID-19 vaccine using the MCDA and to rank the COVID-19 vaccine using evidence before and after approval.

## 2. Materials and Methods

We quantitatively evaluated the benefits and risks of four COVID-19 vaccines (mRNA-1273, BNT162b2, Ad26.COV.2, and ChAdOx1-S) using MCDA and ranked the COVID-19 vaccines using evidence before and after approval. Quantitative BR assessment for four vaccines was performed based on the supporting literature on safety and efficacy of these vaccines before and after approval, and BR profiles were compared. The MCDA model was applied for the quantitative BR assessment of COVID-19 vaccines. We assessed two mRNA-based vaccines and two viral vector-based vaccines that were approved worldwide during the early pandemic: the mRNA-based vaccines BNT162b2 (Pfizer-BioNTech) and mRNA-1273 (Moderna) and viral vector-based vaccines ChAdOx1-S (AstraZeneca) and Ad26.COV2.S (Janssen). COVID-19 vaccine approval information is provided in Table S1.

**Figure 1:**
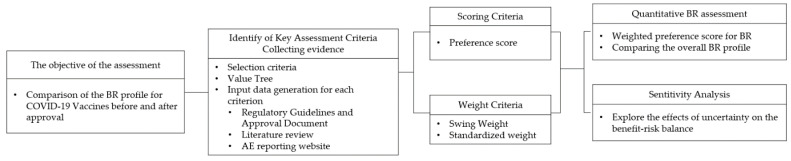
Overview of MCDA process for the benefit-risk assessment model for COVID-19. BR: benefit-risk. MCDA: multi-criteria decision analysis.

To assess the COVID-19 vaccines quantitatively, the following steps were followed: establishing the objective of the assessment; identification of key benefits and risks in a value tree; selection and extraction of relevant data (effect table); scoring and weighting; evaluation of the BR; and sensitivity analyses [12]. An overview of the MCDA process for the COVID-19 benefit-risk assessment model is shown in Figure 1, and each step is described in the following section.

### 2.1. Criteria Selection

We reviewed the endpoints of the clinical trials to select the criteria for this study. Clinical trials for the COVID-19 vaccine were performed according to WHO guidelines, and most clinical trials had the same endpoints for safety and effectiveness [13]. The candidates for benefit criteria were collected from efficacy endpoints in clinical trials and the effectiveness reported in observational studies. The recommended efficacy endpoints in COVID-19 clinical trials were as follows: the number of virologically confirmed symptomatic cases of COVID-19, efficacy versus placebo for COVID-19 prevention, efficacy against severe and non-severe COVID-19, seroconversion rates, antibody quantification and immunogenicity of the booster, and prevention of variants (such as Omicron) [14]. We collected candidates for the risk criteria from the clinical trials. In COVID-19 clinical trials, the following parameters were considered: solicited local and systemic adverse events, unsolicited adverse events, serious adverse events, including death, evidence of adverse events of special interest, and medically attended adverse events [13,15].

**Figure 2:**
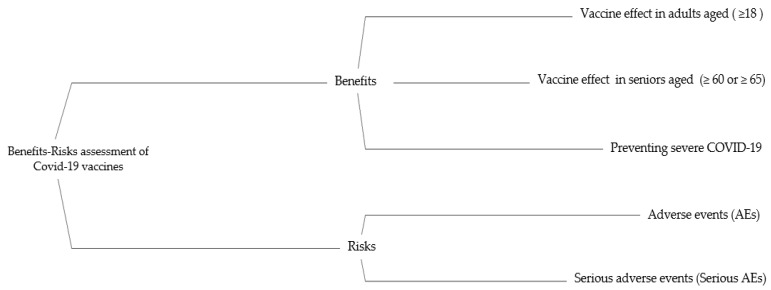
The effects tree for benefit-risk balance of COVID-19 Vaccines. AE: adverse event.

After collecting candidates, the primary and secondary endpoints for safety and efficacy were selected as the criteria for MCDA. Three criteria were chosen as benefit criteria: (1) prevention of COVID-19 infection in adults over the age of 18; (2) prevention of severe COVID-19; and (3) prevention of COVID-19 infection in seniors (60 or older). Two were chosen as the risk criteria: (1) adverse events, including solicited and unsolicited; and (2) serious AEs. The specific descriptions for each criterion are listed in Table 1. The effects tree organizing benefit/risk criteria is shown in Figure 2.

### 2.2. Preference Score

For pre-authorization benefit and risk criteria, input data were retrieved from clinical trials searched in MEDLINE and regulatory documents submitted for emergency use approval (BNT162b2 [16,17], mRNA-1273 [18,19], ChAdOx1-S [20,21], and Ad26.COV2.S [22,23]) (detailed in Tables S2 and S3).

For post-authorization, each criterion input data were gathered using post-authorization studies for benefit criteria and the country-specific adverse event monitoring system for risk criteria. The input data for benefits criteria, a systematic literature search for observational studies after post-authorization, was performed in Scopus, PubMed via Medline, and Google Scholar, as well as the Preprint servers including medRxiv for studies related to the keywords selected based on the Medical Subject Headings (MeSH), published until 30 June 2022. The search was performed using the following keywords by combining MeSH and Non-MeSH terms: “COVID-19 vaccine”, “SARS-CoV-2 coronavirus vaccines”, “BNT162b2”, “mRNA-1273”, “ChAdOx1-S”, “Ad26.COV2.S”, “effectiveness”, “post-vaccination”, “observational studies”, “real-world”. We selected studies that examined the effectiveness of COVID-19 vaccination in adults, hospitalization, and mortality after COVID-19 vaccination and retrieved input data for each criterion. Studies that did not specify the number of participants in both the vaccinated and unvaccinated groups were excluded. Considering the studies examined, people who had not taken any vaccines were classified as unvaccinated, and those who were on the ≥7 days or ≥14 days after a full dose of vaccination were classified as fully vaccinated. Patients in the partial vaccination group were excluded from this study.

Sixteen cohort studies and one phase 3B study were selected, and data were extracted for post-authorization benefit criteria. The characteristics of eligible studies for the post-authorization benefit criteria are shown in Table S4. Among the 17 studies, the data extracted for effectiveness were selected from 13 studies of BNT162b2 [24,25,26,27,28,29,30,31,32,33,34,35,36], seven studies of mRNA-1273 [28,30,32,33,34,35,37], three studies of ChAdOx1-S [25,30,31] and seven studies of Ad26.COV.2 [25,28,34,35,38,39,40]. The vaccine effect (VE) for each criterion was assessed using pooled risk ratios (RRs) for the number of COVID-19 cases or the number of symptomatic and asymptomatic patients after full vaccination (Figures S1–S3). VE was calculated using the formula (1 − RR) × 100%. VE for each criterion for vaccines is provided in Table S5.

For the risk criteria values for post-authorization, the total number of reported cases of AEs and serious AEs after full vaccination was collected from 13 countries that provided a country-specific adverse event monitoring system with vaccination rates of more than 70% [41] (Table S6). We used the safety data for the US that were extracted from a published article for evaluation of safety using the Vaccine Adverse Effect Reporting System (VAERS) data from 14 December 2020 to 30 September 2021 [42]. The county list with the cut-off date, as well as their safety information reporting website, is provided in Table S7.

The input data for each criterion used in our model are summarized in Table S8. Each criterion was measured using a different scale. The input data for each criterion were transformed into a preference score on the same scale from 0 to 100 by a linear value function. The top of the scale was the preferred option and the bottom of the scale was the least preferred option. A fixed scale was used to assess the preference score for each criterion of a given vaccine. For the relative reduction percentage of the benefit effect, a larger input metric received a higher preference score, whereas for the risk effects, a higher percentage or incidence resulted in a lower preference score. As a result, a high-risk criterion score indicated better safety. The preference score for benefit criteria and benefit was calculated using Equations (1) and (2), where *V_ij_* is the preference score of vaccine *j* for the *i*^th^ criterion, and *a_ji_* is the input data of *j* for the *i*^th^ criterion. *C_imax_* and *C_imin_* are the reference values for low and high reference points, respectively. The elicited preference scores are shown in Table 2.
For benefit$\;Preference\;score\;\left(V_{ij}\right)=100\times \left(a_{ji}-C_{imin}\right)/\left(C_{imax}-C_{imin}\right)$(1)For risk$Preference\;score\;\left(V_{ij}\right)=100\times \left(C_{imax\;\;}-a_{ji}\right)/\left(C_{imax}-C_{imin\;\;}\right)$(2)

The preference score represents the strength of preference or performance of each vaccine on each of the five effect scales. The preference score based data collected were calculated using Hiview3 software (Catalyze Ltd., Hursley, UK). The preference score for each criterion was used to calculate a weighted preference score by applying its weight.

### 2.3. Weighting

Weighting was assigned for each criterion. Each criterion was weighted by Korean professionals, including an infectious disease physician and a pharmaceutical company employee developing the COVID-19 vaccine in South Korea. For weighing, candidates among professionals were physicians of infectious disease, who worked in university hospitals, investigators of clinical trials for COVID-19 vaccines, or employees who worked in pharmaceutical companies related to the COVID-19 vaccine. The investigator or member information was procured from the Ministry of Food and Drug Safety in South Korea or the Korean Society of Infectious Diseases, Altogether, 54 physicians were asked to participate in the survey and 16 responded to the survey. Six employees working at COVID-19 vaccine-related pharmaceutical companies also responded to the survey. A total of 22 professionals weighted each criterion using swing weighting: 16 physicians (including 14 infectious disease physicians) and 6 professional employees in the industry. Six of the 14 infectious disease physicians were investigators of COVID-19 clinical trials in South Korea. The demographic characteristics of the professionals who weighed the criteria are shown in Table S9.

The professional compared the relative weights of the most important criterion to determine the weights of another criterion. Swing weighting is a method for determining weighting factors indirectly by comparing criteria against the one deemed most important [43]. The professionals were asked to select the most important benefit criterion as a reference benefit criterion (weight 100). Given a fixed reference benefit criterion, professionals were asked to estimate and select either the least or most important criterion as a reference criterion and assess how much more or less important the other criteria were with respect to the reference benefit criterion. The other weighted criteria were assigned from 0 to 100 based on a comparison with the reference benefit criterion. For the risk criteria, the weighting process was the same as that for the benefit criteria. After collecting weights from professionals, the final step of the swing process was to calculate standardized weights by normalizing each criterion weight against the total weight among all criteria. The standardized weight (W_i_) was calculated using the following Equation (3), where f_i_ is the weight assessed for the i^th^ criterion and i = 1 to n for the number of criteria.



$W_{i}=\frac{f_{i}}{\sum_{i=1}^{n}\mathrm{fi}}$

 all Standardized weight (Wi) sum to 1 

$\overset{n}{\underset{i=0}{\sum }}W_{i}=1$

(3)

The standardized weight for each criterion was summed to provide an overall index of the benefit-risk ratio. HiView3 calculated the standardized weights and displayed the results. The standardized weights for each criterion, as determined by swing weighting, are presented in Table S10. The relative model weights for benefits and risks were 0.609 and 0.391, respectively.

### 2.4. Base case and Sensitivity Analyses

Using a preference score and standardized weight for each criterion, a quantitative BR assessment model was generated using the Hiview3 software to calculate the weighted preference value for each criterion. The BR score was calculated as the sum of the weighted preference scores for each vaccine using the following Equation (4), where W_i_ is the standardized weight and V_ij_ is the preference score of vaccine j of the i^th^ criterion.

(4) $$\mathrm{BR}\;\mathrm{score}\;\left(X\right)=\overset{i=n}{\underset{i=0}{\sum }}W_{i}{*V}_{\mathrm{ij}}$$

The BR score ranged from 0 to 100, with a score of 100 indicating the maximum value in this model. The highest BR score for a vaccine was recommended as the best option. The BR score included all criteria for the benefit and risk values. Therefore, we compared the benefit-to-risk for each vaccine to determine a benefit-risk balance based on subgroup values for benefit and risk. The benefit and risk values are plotted on the map. An ideal option with a high value of benefit and high risk value lies in the top right-hand corner of the map. Criterion contribution analyses were performed to determine the contribution of each criterion to the BR score. The BR score ranked vaccination options based on the risk-benefit score and compared the specific risk and benefit criteria between these vaccines.

Sensitivity analyses were performed to determine the robustness of the findings. Sensitivity analyses were performed within the main model to determine the extent to which the choice of weights (decreased or increased) for benefits and risks affected the outcome of the BR model.

## 3. Results

**Figure 3:**
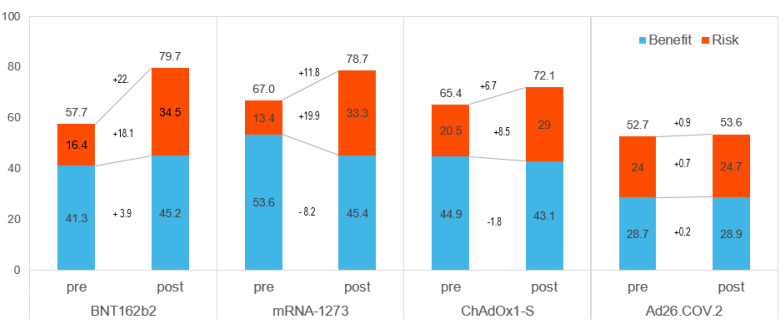
The BR score for each vaccine. Pre: Pre-authorization, Post: Post-authorization.

Table 3 and Figure 3 show the BR scores for each vaccine and the weighted preference scores for each criterion. The BR score for each vaccine was a sum of the weighted preference scores for its criterion. A weighted preference score for each criterion is calculated by multiplying the weights and those preference scores. The BR scores for pre-authorization were 67.0 for mRNA-1273, 65.4 for ChAdOx1-S, 57.7 for BNT162b2, and 52.7 for Ad26.COV.2. Although Ad26.COV.2 had a high-risk score in the pre-authorization model, it was ranked 4th. After approval, the ranking of the COVID-19 vaccine changed. The expression of BNT162b2 was the highest after approval, followed by mRNA-1273. During the post-authorization period, BNT162b2 and mRNA-1273 were 79.7 and 78.7, respectively. The BR scores were 72.1 and 53.6 for ChAdOx1-S and of Ad26.COV.2, respectively. BNT162b2 and mRNA-1273 had high scores for benefits and risks in the post-authorization model.

As shown in Figure 3, the differences between pre- and post-authorization were higher for BNT162b2 and mRNA-1273. The risk score increased considerably for all but Ad26.COV. It was increased to 19.9 for mRNA-1273, 18.1 for BNT162b2, and 8.5 for ChAdOx1-S while Ad26.COV.2 increased by only 0.7 points. After approval, the benefit scores of BNT162b2 and Ad26.COV.2 rose (3.9 and 0.2, respectively), but those of mRNA-1273 (−8.2) and ChAdOx1-S (−1.8) did not.

**Figure 4:**
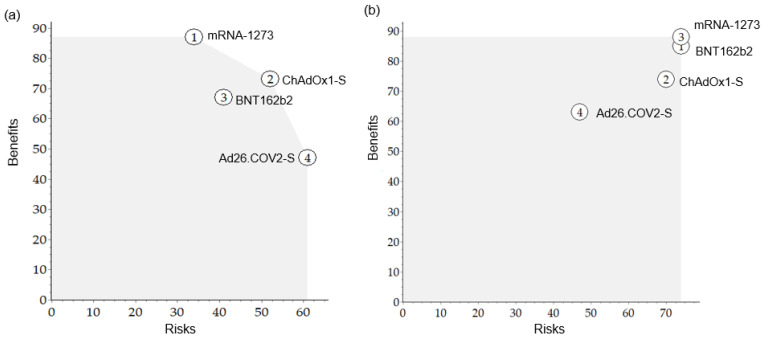
The map of benefits versus risks for each vaccine. (**a**) Pre-authorization, (**b**) Post-authorization.

To compare the benefit-to-risk for each vaccine to see a BR balance, the benefit versus risk was plotted on a map (Figure 4). In the pre-authorization model, ChAdOx1-S showed an overall good benefit-risk balance (located at the top right-hand corner), but its BR score was lower than that of mRNA-1273, which was the most preferred. After authorization, the ideal options in the map were BNT162b2 and mRNA-1273.

**Figure 5:**
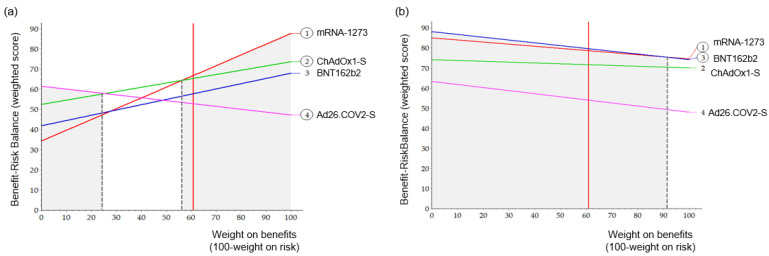
Sensitivity analysis for weight. A vertical black dashed line indicated the point of the preferred vaccine change. (**a**) Pre-authorization, (**b**) Post-authorization.

As a sensitivity analysis, Figure 5 shows the BR score based on the weight of the benefit. This was done for both the pre- and post-authorization models. The vertical red line indicates the current standardized weight of the benefit. The line for each option shows how this total weighted preference score changed depending on the weight of the benefit. The most preferred option at any standardized weight had the highest Y value. In the pre-authorization model, mRNA-1273 was the most preferred vaccine, with a benefit weight of 57 or over. Ad26.COV.2 was the most preferred below 25 for benefit weight, whereas in the range of 26 to 56, ChAdOx1-S was the most preferred. In the post-authorization models, mRNA-1273 was the most preferred in the range of benefit weight of 90 or over, and BNT162b2 was the most preferred in the rest of the range.

## 4. Discussion

We conducted a quantitative BR assessment of COVID-19 vaccines using the MCDA and showed the rank of preferred vaccines based on evidence before and after approval. Before approval, mRNA-1273 ranked the highest, followed by ChAdOx1-S, BNT162b2, and Ad26.COV.2. After approval, BNT162b2 ranked the highest, followed by mRNA-1273, with a 1.0-point difference. The ranking of COVID-19 vaccines changed after approval. mRNA-based vaccines (mRNA-1273, BNT162b2), with high BR scores for both benefits and risks, shifted to the ideal position. In particular, the contribution of the risk increased after approval, except for Ad26.COV.2. Sensitivity analysis showed that mRNA-based vaccines were the preferred vaccines for any change in weight for benefit and risk in post-authorization.

The increase in the benefit/risk score did not mean an actual increase/decrease in benefit or risk, because the scores before and after approval were obtained from different data sources, especially the study design (RCT or an observational study). However, it led to a relative rank change. In the case of BNT162b2, the severe COVID-19 score was significantly lower than that of other vaccines preapproval, but the score improved after receiving the data in post-authorization. In terms of benefit, only the mRNA-1273 score decreased dramatically. mRNA-1273 was ranked first with a relatively large difference in preauthorization, but it was scored at a level similar to that of others in post-authorization. Differences in the benefits of mRNA-1273 were highest in VE aged 18 or over and severe COVID-19. Due to the approval of the COVID-19 vaccines for conditional or emergency use in the pandemic, evidence was limited, including a lack of data on hospital admissions and deaths at the time of conditional approval [44]. As shown in the results, the rank of the vaccines changed depending on the evidence collected during different periods. The case of mRNA-1273 shows the need to derive robust results through accumulated information updates. This also demonstrated the potential for additional real-world evidence to be utilized in regulatory science. Since the latest approval date of mRNA-1273 was March 2021 (EMA), we collected data on additional benefits and risks for at least one year after approval. Although there might be a publication lag in reporting benefits, adverse event rates from the reporting system can be considered the most up-to-date and cumulative information. When only pre-approval information was used, the rank was not robust, depending on the weights, whereas that in the post-approval period was robust. This shows that updated post-marketing benefits and risks should be monitored after approval for emergency use from the perspective of regulatory decisions. In addition, a change in rank indicates that restricted information can affect the optimal use of products.

On the other hand, the FDA uses a qualitative benefit-risk framework and works as a flexible mechanism for communicating important decision factors, allowing it to support the diversity of drug approval decisions. In contrast, EMA focuses on quantitative benefit-risk assessment [45]. A previous study highlighted the flexibility of the FDA’s framework [46]. In addition to the qualitative benefit-risk framework, quantitative BR assessment also needs to be flexible. Our model also demonstrates the possibility of flexibly updating a quantitative model.

To the best of our knowledge, no previous studies have assessed vaccines using MCDA; therefore, it is difficult to compare the results directly with those of previous studies. Instead, we have confirmed the criteria for each vaccine. In a previous network meta-analysis, mRNA-1273 and BNT162b2 were significantly better than placebo in VE, whereas the effect of Ad26.COV.2. was not significant [47]. This was consistent with the trend in the benefit scores in this study. However, the serious AEs of all vaccines were not statistically significant, although we did not consider statistical significance against placebo. Beyond collecting data, network meta-analysis, in which multiple treatments are being compared, can be used for advanced analysis. In contrast, real-world data showed that mRNA-based vaccines were comparable to or had better safety profiles than viral-vector vaccines [48]. The results of comparing the two mRNA vaccines showed that the incidence of AEs and serious AEs for BNT162b2 was slightly lower than that for mRNA-1273 [49]. Therefore, the outcomes of the quantitative BR model for both pre- and post-authorization in the study were consistent with the published literature results [48,50].

Our results should be interpreted cautiously in the context of the following aspects: The effectiveness of SARS-CoV2 variants was not considered, although there might be differences among the vaccines. We considered the inclusion of VE against variants; however, there was a lack of concrete information on variants for each vaccine. The literature selected in the study for the input data did not provide variant effect for the vaccine. It was not adequate to apply the pre-authorization model because of a lack of information. Thus, the variants criterion was not included in the value tree. In addition, the level of VE against the variants varied depending on previous types of vaccination. Therefore, it was not applied to the post-authorization model because we could not independently estimate the vaccine effect by itself. If the effect of vaccines on variants is confirmed, it can be reflected in BR assessments in further studies. Weight cannot be generalized to the general population because we only surveyed professionals. Specifically, preferences can differ between physicians and industry employees; however, we did not consider weight according to stakeholders. However, we conducted a sensitivity analysis and found a robust rank regardless of the weight.

The input data we retrieved were mostly from the published literature, except for safety information for post-approval. Post-approval data on the number of AEs and serious AEs were collected from a country-specific AE reporting website containing publicly accessible information. Most safety-reporting systems are self-reporting systems. Therefore, the collected data had the potential to be biased. However, Singh et al. showed that the frequency of side effects for BNT162b2 and mRNA-1273 was significantly lower than that for Ad26.COV.2 after vaccination, based on Vaccine Adverse Effect Reporting System (VAERS) data [51]. Moreover, the incidence of AEs or serious AEs following Ad26.COV.2 vaccination was 1.8 to 3.7 times higher than that following BNT162b2 or mRNA-1273 vaccination [42,49]. In several published studies, the side-effect rate for COVID-19 vaccines was similar to the data we collected.

Statistical analysis for BR scores between vaccines could not be performed because the BR score for each vaccine was one figure. Because we did not perform statistical analysis, we could not confirm that the difference between mRNA-1273 and BNT162b was significant. Although BNT162b2 was the most preferred in majority of the range post-authorization in a sensitivity analysis, it cannot be interpreted as superiority of BNT162b2. The result should be interpreted to suggest that mRNA-based vaccines (mRNA-1273, BNT162b2) were robustly placed in the ideal position with high BR scores for both benefits and risks, regardless of the balance of benefits and risk.

Despite these limitations, this study was the first to rank vaccines based on BR scores and analyze the benefits and risks of COVID-19 vaccines. We developed a quantitative BR assessment model and incorporated effectiveness and uncertainty into models reflecting the weights of healthcare professionals. This model can be used for future studies related to health technology assessment, assessing COVID-19 vaccines by accumulating live information on risks and benefits. Unless the evaluation criteria change, this model can be applied to the quantitative BR assessment for COVID-19 vaccines in terms of global or country-level data.

## 5. Conclusions

The MCDA is useful for comparing medicinal products by considering multiple benefits and risks, various data sources, and stakeholder preferences in a single model. We demonstrated a quantitative BR assessment using MCDA and provided the rank of COVID-19 vaccines, focusing on the evidence gap between the time of approval and post-approval. We found that mRNA-based vaccines were preferred before and after approval. This model can be used for future studies related to health technology assessment, assessing COVID-19 vaccines by accumulating live information on risks and benefits.

## Figures and Tables

**Table 1:** Definitions of the benefit and risk criteria.

Criteria		Description
Benefits	Vaccine effect in adults aged ≥18	Vaccine efficacy to prevent the occurrence of COVID-19 in adult (age ≥ 18) after full dose of vaccines
	Vaccine effect in seniors aged ≥60 or ≥65	Vaccine efficacy to prevent the occurrence of COVID-19 in people aged 60 and over (or age ≥ 65) after full dose of vaccines
	Preventing severe COVID-19	Vaccine efficacy to prevent severe COVID-19 Severe COVID-19: severe systemic illness with medical care including death
Risks	Adverse events	Adverse events including solicited (local and systemic) and unsolicited AEs after vaccination
	Serious adverse events	Serious adverse events including any medical complication that, at any dose, poses a risk of death is life-threatening, necessitates hospitalization or an extension of hospitalization, causes persistent or severe disability, or impairment of function

**Table 2:** Preference score of the benefit and risk criteria for each vaccine.

Criteria	Pre-Authorization				Post-Authorization			
	BNT162b2	mRNA-1273	ChAdOx1-S	Ad26.COV.2	BNT162b2	mRNA-1273	ChAdOx1-S	Ad26.COV.2
Benefits								
VE, age ≥ 18	90.0	88.2	48.0	33.8	80.0	74.0	44.0	42.0
VE, age ≥ 60 (or 65)	89.4	72.8	67.0	52.6	64.0	66.0	66.0	42.0
Severe COVID-19	32.8	100.0	100.0	53.4	78.0	82.0	96.0	56.0
Risks								
AEs	41.6	33.5	51.9	60.5	86.5	78.4	71.8	49.1
Serious AEs	42.0	35.0	53.0	62.0	89.3	89.7	75.8	72.9

**Table 3:** The benefit-risk score.

Criteria	Weight	Pre-Authorization				Post-Authorization			
		mRNA-1273	ChAdOx1-S	BNT162b2	Ad26.COV.2	mRNA-1273	ChAdOx1-S	BNT162b2	Ad26.COV.2
BR score (rank)		67.0 (1)	65.4 (2)	57.7 (3)	52.7 (4)	78.7 (2)	72.1 (3)	79.7 (1)	53.6 (4)
Benefits (rank)		53.6 (1)	44.9 (2)	41.3 (3)	28.7 (4)	45.4 (1)\	43.1 (3)	45.2 (2)	28.9 (4)
VE, age ≥ 18	0.188	16.6	9.0	16.9	6.4	13.9	8.3	15.0	7.9
VE, age ≥ 60 (or 65)	0.188	13.7	12.6	16.8	9.9	12.4	12.4	12.0	7.9
Severe COVID-19	0.233	23.3	23.3	7.6	12.4	19.1	22.4	18.2	13.1
Risks (rank)		13.4 (4)	20.5 (2)	16.4 (3)	24.0 (1)	33.3 (2)	29.0 (3)	34.5 (1)	24.7 (4)
AEs	0.158	5.3	8.2	6.6	9.5	12.4	11.3	13.7	7.7
Serious AEs	0.233	8.1	12.3	9.8	14.5	20.9	17.7	20.8	17.0

## Data Availability

Not applicable.

## Supplementary Materials

The following supporting information can be downloaded from https://www.mdpi.com/article/10.3390/vaccines10122029/s1, (Table S1) COVID-19 Vaccine approval by the regulatory authority, (Table S2) Input data for the benefit criteria for pre-authorization. (Table S3) Input data for the risk criteria for pre-authorization, (Table S4) Characteristics of eligible studies for post-authorization benefit criteria, (Table S5) Input data for the benefit criteria for post-authorization, (a) BNT162b2, (b) mRNA-1273, (c) ChAdOx1-S, and (d) Ad26.COV2.S, (Table S6) Input data for risk criteria for post-authorization, (Table S7) List of country-specific adverse event reporting sites for post-authorization risk criteria (13 countries). (Table S8) Summary of input data for benefit and risk criteria for pre- and post-authorization, (Table S9) Demographic characteristics of professionals to weigh the criteria, (Table S10)Weight and standardized weight of criteria, (Figure S1) Results of meta-analysis: Vaccine effect in adults aged ≥18 years for post-authorization, (a) BNT162b2, (b) mRNA-1273, (c) ChAdOx1-S, and (d) Ad26.COV2.S, (Figure S2) Results of meta-analysis: Vaccine effect in seniors aged (≥60 or ≥65) for post-authorization, (a) BNT162b2, (b) mRNA-1273, (c) ChAdOx1-S, and (d) Ad26.COV2.S, (Figure S3) Results of meta-analysis: Preventing severe COVID-19 post-authorization, (a) BNT162b2, (b) mRNA-1273, (c) ChAdOx1-S, and d) Ad26.COV2.S.
