# Supplementary material for: Quantitative Benefit–Risk Assessment of COVID-19 Vaccines Using the Multi-Criteria Decision Analysis
Source: Vaccines (Basel). 2022 Nov 27;10(12):2029. doi: 10.3390/vaccines10122029 (PMC9785565; doi:10.3390/vaccines10122029)

Supplementary material

Quantitative Benefit–Risk Assessment of COVID-19 Vaccines Using the Multi-Criteria Decision Analysis

Kyung-Hwa Son †, Sun-Hong Kwon †, Hye-Jung Na, Youngsuk Baek, Inok Kim and Eui-Kyung Lee *

School of Pharmacy, Sungkyunkwan University, Suwon 16419, Republic of Korea
* Correspondence: ekyung@skku.edu
† These authors contributed equally to this work.

These materials have been provided by the authors to provide readers with additional information regarding their work.

Table: List of contents

Table S1. COVID-19 Vaccine approval by the regulatory authority. 3

Table S2. Input data for benefit criteria for pre-authorization........ 4

Table S3. Input data for risk criteria for pre-authorization. 4

Table S4. Characteristics of eligible studies according to post-authorization benefit criteria. 5

Table S5. Input data for the benefit criteria for post-authorization, a) BNT162b2, b) mRNA-1273, c) ChAdOx1-S, and d) Ad26.COV2.S 6

Table S6. Input data for risk criteria for post-authorization. 7

Table S7. List of country-specific adverse event reporting sites for post-authorization risk criteria (13 countries). 8

Table S8. Summary of input data for benefit and risk criteria for pre- and post-authorization. 9

Table S9. Demographic characteristics of professionals to weigh the criteria. 10

Table S10. Weight and standardized weight of the criterion. 10

Figure: List of contents.

Figure S1. Results of meta-analysis: Vaccine effect in adults aged ≥18 years for post-authorization, a) BNT162b2, b) mRNA-1273, c) ChAdOx1-S, and d) Ad26.COV2.S........ 11

Figure S2. Results of meta-analysis: Vaccine effect in seniors aged (≥ 60 or ≥ 65) for post-authorization, a) BNT162b2, b) mRNA-1273, c) ChAdOx1-S, and d) Ad26.COV2.S........ 11

Figure S3. Results of meta-analysis: Preventing severe COVID-19 post-authorization, a) BNT162b2, b) mRNA-1273, c) ChAdOx1-S, and d) Ad26.COV2.S 12

Table S1. COVID-19 Vaccine approval by the regulatory authority.

Vaccine platform description	Vaccine	Commercial name	Developer	US Food and Drug Administration (FDA)[a]	European Medicines Agency (EMA)[b]	Approval country*	WHO Emergency Use listing
RNA-based vaccine	BNT162b2	Comirnaty	Pfizar/BioNTech	2020-12-11<br>2021-08-23[c]	2020-12-21	149 countries	yes
RNA-based vaccine	mRNA-1273	Spikevax	Moderna	2020-12-18	2021-03-11	88 countries	yes
Viral vector(non-replicating)	ChAdOx1-S	Vaxervira	AztraZeneca	na	2021-01-11	149 countries	yes
Viral vector(non-replicating)	Ad26.COV.2	Jcovden	Jansen Pharmaceutical	2021-02-27	2021-02-16	113 countries	yes

a: emergency use authorization (EUA) by the US Food and Drug Administration (FDA)
b: conditional marketing authorization by the European Medicines Agency (EMA) and the UK Medicines and Healthcare products Regulatory Agency, c: standard review authorization, na: not applicable *https://covid19.trackvaccines.org/ (accessed on 9 Sep 2022)

Table S2. Input data for benefit criteria for pre-authorization.

Vaccines	Source	Vaccine effect in adults aged (≥18)	Vaccine effect in seniors aged (≥ 60 or ≥ 65)	Preventing severe COVID-19
		VE (%, 95% CI)	VE (%, 95% CI)	VE (%, 95% CI)
BNT162b2	EMA	95 (90.3, 97.6)	94.7 (66.7, 99.9)	66.4 (-124.8, 96.3)
mRNA-1273	FDA	94.1 (89.3, 96.8)	86.4 (61.4, 95.5)	100 (887.0, NE)
ChAdOx1-S	Phase 3 (Falsey et al.[21])	74.0 (65.3, 80.5)	83.5(54.2-94.1)	100.0 (71.6, NE)
Ad26.COV2.S	FDA	66.9 (59.0, 73.4)	76.3 (61.6, 86.0)	76.7 (54.6, 89.1)

BNT162b2 (Pfizer-BioNTech), mRNA-1273 (Moderna), ChAdOx1-S (AstraZeneca), Ad26.COV2.S (Janssen); EMA, European Medicines Agency; FDA, US Food and Drug Administration; VE, vaccine effect.

Table S3. Input data for risk criteria for pre-authorization.

Vaccines	Source	Adverse Events			Adverse Events AVG %	Serious Adverse Events (%)
		Solicited (Local) %	Solicited (Systemic) %	Unsolicited (local/Systemic) %		
BNT162b2	FDA	73.8	71.3	30.2	58.4	0.58
mRNA-1273	Phase 3 (El Sahly et al.[18])	88.7	79.5	31.3	66.5	0.65
ChAdOx1-S	EMA, Phase 3 (Falsey et al. .[21])	58.6	56.0	29.7	48.1	0.47
Ad26.COV2.S	FDA, Phase 3 (Sadoff et al.[22])	50.3	55.1	13.1	39.5	0.38

Table S4. Characteristics of eligible studies according to post-authorization benefit criteria.

Vaccine	Author (year)	Data collection period		Study design	Location	Study group	Participants	
		From	To				Vaccinated	Unvaccinated
Ad26.COV2.S	Arregocés-Castillo (2022) [25]	2021-03-11	2021-10-26	cohort study	Colombia	≥60	730863	1414147
Ad26.COV2.S	Bekker (2022) [38]	2021-02-17	2021-05-17	Phase 3 B	South Africa	≥18	215813	215813
Ad26.COV2.S	Corchado-Garcia (2021) [39]	2021-02-27	2021-07-22	cohort study	USA	≥18	8880	88627
Ad26.COV2.S	Grannis (2021) [28]	2021-06-01	2021-08-01	cohort study	US	≥18	390517	1524153
Ad26.COV2.S	Polinski (2021) [40]	2021-03-01	2021-07-21	cohort study	US	≥18, ≥60	44573	43228
Ad26.COV2.S	Rosenberg (2022) [34]	2021-05-01	2021-08-31	cohort study	US	≥18, ≥65	5638142	3052683
Ad26.COV2.S	Thompson (2021) [35]	2020-12-14	2021-04-10	cohort study	USA	≥18	15581	20406
BNT162b2	Angel (2021) [24]	2020-12-20	2021-02-25	cohort study	Israel	≥18	5372	696
BNT162b2	Arregocés-Castillo (2022) [25]	2021-03-11	2021-10-26	cohort study	Colombia	≥60	730863	1414147
BNT162b2	Britton (2021) [26]	2020-12-21	2021-02-21	cohort study	USA	≥18	304	87
BNT162b2	Fabiani (2021) [27]	2020-12-27	2021-03-24	cohort study	Italy	≥18	5186	1090
BNT162b2	Grannis (2021) [28]	2021-06-01	2021-08-01	cohort study	US	≥18	15035	17832
BNT162b2	Hall (2021) [29]	2020-12-07	2021-02-05	cohort study	UK	≥18	1605	2683
BNT162b2	Martínez-Baz (2021) [30]	2021-01-01	2021-04-01	cohort study	Spain	≥18, ≥60	1036	19580
BNT162b2	Menni (2021) [31]	2021-12-08	2021-03-10	cohort study	UK	≥18	103622	464356
BNT162b2	Paris (2021) [32]	2021-01-04	2021-05-17	cohort study	France	≥18	2042	3573
BNT162b2	Pawlowsk (2021) [33]	2020-12-01	2021-04-20	cohort study	USA	≥18	44573	43228
BNT162b2	Rosenberg (2022) [34]	2021-05-01	2021-08-31	cohort study	US	≥18, ≥65	5638142	3052683
BNT162b2	Thompson (2021) [35]	2020-12-14	2021-04-10	cohort study	USA	≥18	15581	20406
BNT162b2	Zacay (2021) [36]	2021-01-01	2021-02-11	cohort study	Israel	≥18	2941	1900
ChAdOx1-S	Arregocés-Castillo (2022) [25]	2021-03-11	2021-10-26	cohort study	Colombia	≥60	730863	1414147
ChAdOx1-S	Martínez-Baz (2021) [30]	2021-01-01	2021-04-01	cohort study	Spain	≥18, ≥60	1036	19580
ChAdOx1-S	Menni (2021) [31]	2021-12-08	2021-03-10	cohort study	UK	≥18	103622	464356
mRNA-1273	Bruxvoort (2022) [37]	2020-12-18	2021-03-31	cohort study	USA	≥18, ≥65	352878	352878
mRNA-1273	Grannis (2021) [28]	2021-06-01	2021-08-01	cohort study	US	≥18	15035	17832
mRNA-1273	Martínez-Baz (2021) [30]	2021-01-01	2021-04-01	cohort study	Spain	≥18	1036	19580
mRNA-1273	Paris (2021) [32]	2021-01-04	2021-05-17	cohort study	France	≥18	2042	3573
mRNA-1273	Pawlowsk (2021)[33]	2020-12-01	2021-04-20	cohort study	USA	≥18	44573	43228
mRNA-1273	Rosenberg (2021) [34]	2021-05-01	2021-08-31	cohort study	US	≥18, ≥65	5638142	3052683
mRNA-1273	Thompson (2021) [35]	2020-12-14	2021-04-10	cohort study	USA	≥18	15581	20406

Table S5. Input data for the benefit criteria for post-authorization, a) BNT162b2, b) mRNA-1273, c) ChAdOx1-S, and d) Ad26.COV2.S

Vaccine	Vaccine effect in adults aged ≥18 years				Vaccine effect in seniors aged ≥ 60 or ≥ 65 years				Preventing severe COVID-19			
	Studies	Participants	Effect Estimate	%(1-RR)	Studies	Participants	Effect Estimate	%(1-RR)	Studies	Participants	Effect Estimate	%(1-RR)
BNT162b2	11	5117783	0.10 [0.07, 0.12]]	90%	3	3157413	0.18 [0.14, 0.24]	82%	6	7729469	0.11 [0.07, 0.17]	89%
mRNA-1273	6	4428138	0.13 [0.08, 0.20]	87%	2	1743039	0.17 [0.09, 0.32]	83%	6	6188643	0.09 [0.07, 0.13]	91%
ChAdOx1-S	2	516663	0.28 [0.10, 0.80]	72%	2	1684003	0.17 [0.02, 1.43]	83%	2	1699981	0.02 [0.01, 0.07]	98%
Ad26.COV2.S	5	4738622	0.29 [0.26, 0.34]	71%	3	2809435	0.29 [0.19, 0.43]	71%	7	7222306	0.22 [0.14, 0.32]	78%

Table S6. Input data for risk criteria for post-authorization.

Vaccines	No. of countries	No. of vaccination	No. of AEs	No. of Serious AEs	Country
BNT162b2	13	975647404	1316862	104221	Australia, Austria, Canada, Denmark Germany, Italy, Japan, New Zealand, Norway, South Korea Sweden United Kingdom, United States
mRNA-1273	12	352854545	762905	36347	Australia, Austria, Canada, Denmark, Germany, Italy, Japan, Norway, South Korea, Sweden, United Kingdom, United States
ChAdOx1-S	12	114282186	322794	27645	Australia, Austria Canada, Denmark Germany, Italy, Japan, New Zealand, Norway, South Korea, Sweden, United Kingdom
Ad26.COV2.S	8	21074362	107225	5714	Australia, Canada, Denmark, Germany Italy, Norway, South Korea, United States

AE, adverse event; No., number

Table S7. List of country-specific adverse event reporting sites for post-authorization risk criteria (13 countries).

Country	Vaccination*	Cut-off Date	Vaccine
Australia	84%	Jun 16 2022	BNT162b2, ChAdOx1-S mRNA-1273
Austria	77%	May 27 2022	Ad26.COV2.S, BNT162b2, ChAdOx1-S, mRNA-1273
Canada	83%	May 13 2022	Ad26.COV2.S, BNT162b2, ChAdOx1-S mRNA-1273
Denmark	82%	May 17 2022	Ad26.COV2.S, BNT162b2, ChAdOx1-S mRNA-1273
Germany	76%	Mar 31 2022	Ad26.COV2.S, BNT162b2, ChAdOx1-S mRNA-1273
Italy	81%	Mar 26 2022	Ad26.COV2.S, BNT162b2, ChAdOx1-S mRNA-1273
Japan	82%	Jun 09 2022	BNT162b2, ChAdOx1-S, mRNA-1273
New Zealand	81%	Apr 30 2022	BNT162b2, ChAdOx1-S
Norway	75%	Jun 16 2022	Ad26.COV2.S, BNT162b2, ChAdOx1-S,mRNA-1273
South Korea	86%	Jun 09 2022	Ad26.COV2.S, BNT162b2, ChAdOx1-S,mRNA-1273
Sweden	73%	Jun 02 2022	BNT162b2, ChAdOx1-S, mRNA-1273
United Kingdom	75%	Jun 8 2022	BNT162b2, ChAdOx1-S, mRNA-1273
United States	68%	Sep 30 2021**	Ad26.COV2.S, BNT162b2, mRNA-1273

*Source: COVID-19 Data Explorer, https://ourworldindata.org/explorers[41], (assessed on 11 Sep 2022)

**collected from published reference (from 2020-12-14 to 2021-09-3, Sa, S., et al. (2022))[42]

Australia(https://www.tga.gov.au/covid-19-vaccines),Austria(https://www.basg.gv.at/en/about-us/statistic), Canada(https://health-infobase.canada.ca/covid-19/vaccine-safety), Denmark(https://laegemiddelstyrelsen.dk/da/nyheder/temaer/indberettede-bivirkninger-ved-covid-19-vaccine) Germany (https://www.pei.de/EN/newsroom/dossier/coronavirus/safety-report-covid-19-vaccines-current.html)

Italy (https://www.aifa.gov.it/en/farmacovigilanza-vaccini-covid-19)

Japan(lhttps://www.mhlw.go.jp/stf/seisakunitsuite/bunya/0000164708_00079.html),

New Zealand (https://www.medsafe.govt.nz/COVID-19/vaccine-report-overview.asp)

Norway (https://legemiddelverket.no/English),

South Korea (https://ncv.kdca.go.kr/board.es?mid=a11707010000&bid=0032),

Sweden(https://www.lakemedelsverket.se/en/coronavirus/covid-19-vaccine),United Kindom (https://www.gov.uk/government/publications/

Table S8. Summary of input data for benefit and risk criteria for pre- and post-authorization.

Criteria	Range (worst-best)	Pre-authorization				Post-authorization			
		mRNA-1273	ChAdOx1-S	BNT162b2	Ad26.COV.2	mRNA-1273	BNT162b2	ChAdOx1-S	Ad26.COV.2
Benefits									
VE (≥18), %	66-95	94.1	74.0	95.0	66.9	87.0	90.0	72.0	71.0
VE(≥60 or≥65), %	71-95	86.4	83.5	94.7	76.3	83.0	82.0	83.0	71.0
Preventing severe COVID-19, %	66-100	100.0	100.0	66.4	76.7	91.0	89.0	98.0	78.0
Risks									
AEs, %	Pre: 67-40<br>Post: 0.5-0.1	66.5	48.1	58.4	39.5	0.216	0.135	0.282	0.509
Serious AEs, %	Pre: 0.7-0.4<br>Post: 0.03-0.01	0.65	0.47	0.58	0.38	0.0103	0.0107	0.0242	0.0271

Table S9. Demographic characteristics of professionals to weigh the criteria.

Variable	Total (n=22)
Sex, n(%)	
Male	16(72.73%)
Female	6(27.27%)
Age, years, n(%)	
30~39	3(13.64%)
40~49	13(59.09%)
≥50	6(27.27%)
Professional Field, n(%)	
Physicians	10(45.45%)
Physicians (investigator in COVID-19 vaccine clinical trial)	6(27.27%)
Industry employee	6(27.27%)

Table S10. Weight and standardized weight of the criterion.

Criteria	The measured weights in survey, median (IQR)	Standardized weight
VE (≥18)	79.6 (64.75, 80)	0.188
VE (≥60 or≥65)	80 (80, 89.1)	0.188
Preventing severe COVID-19	99 (89.32, 100)	0.233
AEs	67 (60.75, 77.5)	0.158
Serious AEs	99 (90, 100)	0.233
Total	425	1

Figure S1. Results of meta-analysis: Vaccine effect in adults aged ≥18 years for post-authorization, a) BNT162b2, b) mRNA-1273, c) ChAdOx1-S, and d) Ad26.COV2.S

a) BNT162b2

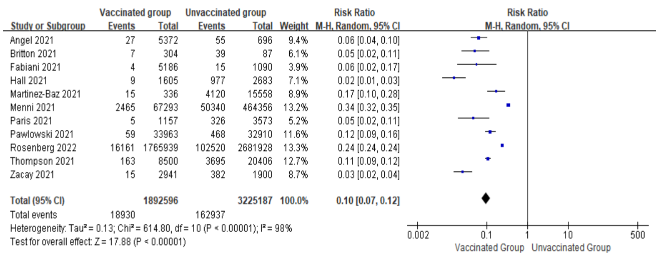

b) mRNA-1273

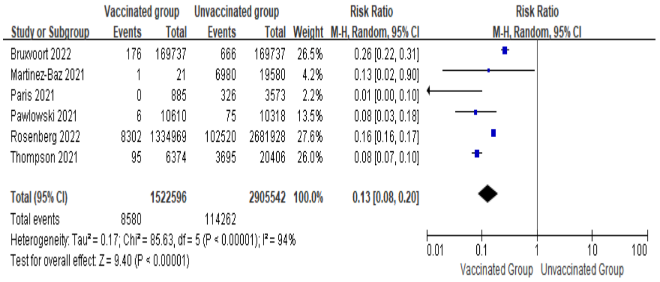

c) ChAdOx1-S

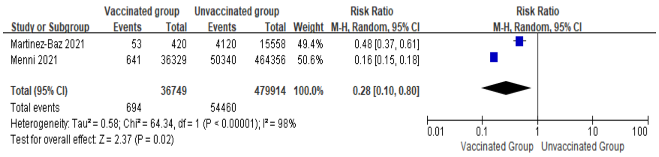

d) Ad26.COV2.S

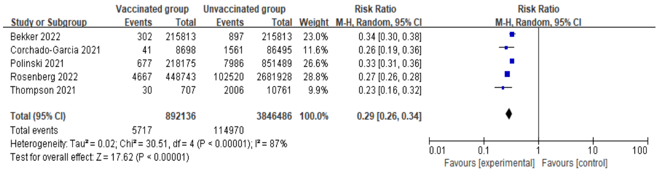

Rectangle (blue): wight size, Rhombus: pooled effect size

Figure S2. Results of meta-analysis: Vaccine effect in seniors aged (≥ 60 or ≥ 65) for post-authorization, a) BNT162b2, b) mRNA-1273, c) ChAdOx1-S, and d) Ad26.COV2.S

a) BNT162b2

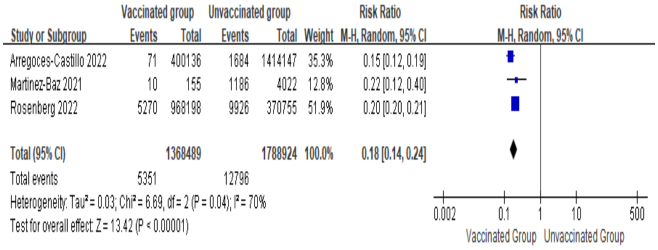

b) mRNA-1273

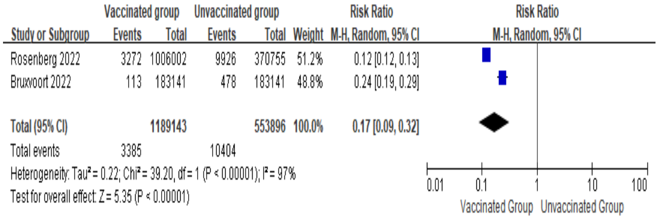

c) ChAdOx1-S

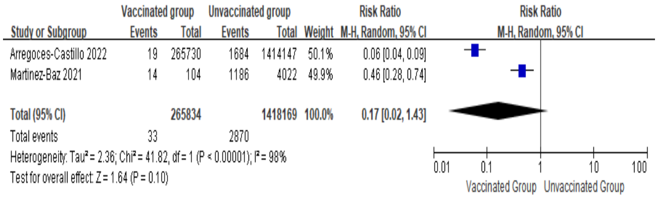

d) Ad26.COV2.S

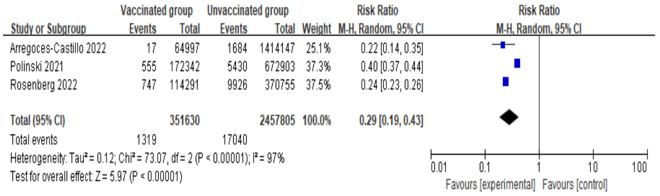

Figure S3. Results of meta-analysis: Preventing severe COVID-19 post-authorization, a) BNT162b2, b) mRNA-1273, c) ChAdOx1-S, and d) Ad26.COV2.S

a) BNT162b2

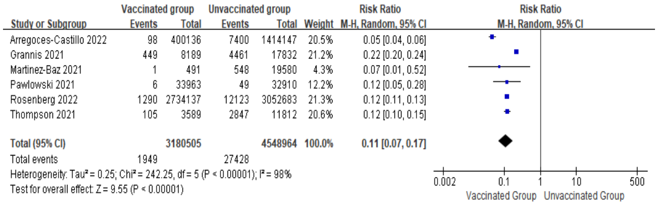

b) mRNA-1273

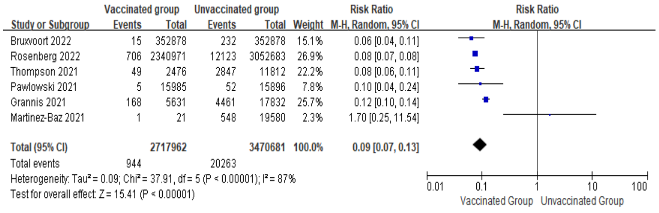

c) ChAdOx1-S

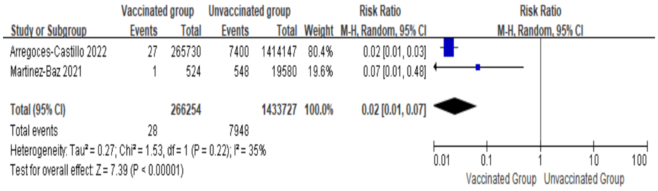

d) Ad26.COV2.S

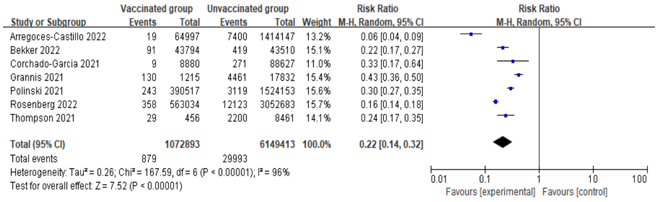

Supplement: Supplementary file 1 [file vaccines-10-02029-s001.zip › vaccines-2005723-supplementary.pdf]
